# Reduced long-term memory in a rat model of 8 minutes ventricular fibrillation cardiac arrest: a pilot trial

**DOI:** 10.1186/s12917-016-0740-6

**Published:** 2016-06-13

**Authors:** Wolfgang Weihs, Alexandra-M Warenits, Florian Ettl, Ingrid A. M. Magnet, Ursula Teubenbacher, Andreas Hilpold, Andreas Schober, Christoph Testori, Akos Tiboldi, Katharina Tillmann Mag, Michael Holzer, Sandra Hoegler, Andreas Janata, Fritz Sterz

**Affiliations:** Department of Emergency Medicine, Medical University of Vienna, Währinger Gürtel 18-20, 1090 Wien, Austria; Department of Pathobiology, University of Veterinary Medicine Vienna, Veterinaerplatz 1, 1210 Wien, Austria; Department of Anaesthesiology, General Intensive Care and Pain Management, Medical University of Vienna, Währinger Gürtel 18-20, 1090 Wien, Austria; Core Center of Biomedical Research, Medical University of Vienna, Währinger Gürtel 18-20, 1090 Wien, Austria; II Medical Department of Cardiology, Hanusch Hospital, Vienna, Heinrich-Collin-Straße 30, 1140 Wien, Austria

**Keywords:** Induced, Cardiac arrest, Cardiopulmonary resuscitation, Rats, Behavioral testing, Morris Water Maze

## Abstract

**Background:**

Evaluating beneficial effects of potential protective therapies following cardiac arrest in rodent models could be enhanced by exploring behavior and cognitive functions. The Morris Water Maze is a well-known cognitive paradigm to test spatial learning and memory.

**Results:**

Behavioral testing with the Morris Water Maze in Sprague–Dawley rats (300 ± 25 g) resuscitated after 8 min of ventricular fibrillation cardiac arrest was carried out 5 and 12 weeks after cardiac arrest (CA) and compared to results of naïve rats (Control).

At 5 weeks, within each group latency time to reach the hidden platform (reflecting spatial learning) decreased equally from day 1 to 4 (CA: 105.6 ± 8.2 vs. 8.9 ± 1.2 s, *p* < *0.001*; Control: 75.5 ± 13.2 vs. 17.1 ± 4.5, *p* < *0.001*) with no differences between groups (*p* = *0.138*). In the probe trial 24 h after the last trial, time spent in the target sector (reflecting memory recall) within each group was significantly longer (CA: 25 ± 1.3; Control: 24.7 ± 2.5 s) than in each of the three other sectors (CA: 7.7 ± 0.7, 14.3 ± 2.5, 8.4 ± 0.8 and Control: 7.8 ± 1.2, 11.7 ± 1.5, 10.3 ± 1.6 s) but with no significantly differences between groups. Seven days later (reflecting memory retention), control group animals remained significantly longer in the target sector compared to every other sector, whereas the cardiac arrest group animals did not. Even 12 weeks after cardiac arrest, the single p values showed that the control animals displayed a trend to perform better than the resuscitated animals.

**Conclusions:**

Memory recall was impaired early after 8 min of ventricular fibrillation cardiac arrest and might be a more valuable tool for cognitive testing than learning recall after global ischemia due to cardiac arrest.

## Background

Despite intensive research, sudden cardiac arrest remains a deadly occurrence with more than 350 000 victims yearly in Europe and the USA [1, 2]. In recent years, animal models have helped to find new therapeutic strategies to reduce injuries from global ischemia and reperfusion following prolonged, untreated cardiac arrest [3–6].

We have recently established a rat ventricular fibrillation cardiac arrest model to investigate pathophysiologic mechanisms of cerebral ischemia and reperfusion. Remarkably, compared to our well-established pig model [7–11], it proved more difficult to generate ventricular fibrillation cardiac arrest survivors with considerable and consistent neurological damage in rats than in pigs with conventional resuscitation therapies. Rats could either be resuscitated with apparently good neurological outcome or did not survive [12]. To generate a target for new therapeutic strategies, it seemed necessary to develop more sophisticated outcome tools to detect subtle neurologic sequelae from cardiac arrest in these seemingly sound rats surviving ventricular fibrillation cardiac arrest. Additional sensitive neurologic and behavioral tests would enhance the ability of such models to evaluate possible beneficial effects of protective therapies. The Morris Water Maze is a very well established paradigm to test spatial learning and memory [13].

Cardiac arrest survivors frequently suffer from sustained memory issues. One of the brain regions responsible for this memory loss is the CA1 region of the hippocampus [14], which is selectively vulnerable to global ischemia [15]. The integrity of hippocampal structure is essential for spatial learning [16]. Hippocampal place cells have been suggested as the primary substrate of spatial memory abilities underlying the spatial navigation process involved in locating the hidden platform in the Morris Water Maze [17, 18]. The Morris Water Maze testing might be a useful additional tool to detect damages caused by systemic ischemia after ventricular fibrillation cardiac arrest and reperfusion injuries occurring during resuscitation and to optimize therapies in small animal models. The aim of this study was to gain preliminary evidence in behavioral testing using Morris Water Maze in rats resuscitated after 8 min of ventricular fibrillation cardiac arrest.

## Methods

A total of 37 adult male Sprague–Dawley rats (300 ± 25 g, 10 weeks of age; Himberg, Austria) were brought to the laboratory 14 days before the experiment, maintained on 12:12 h light/dark cycle with ad libitum access to water and feed and adapted to the new environment. Those eligible for Morris Water Maze testing after the insult (Cardiac Arrest [CA]: *n* = 12) were compared to 10 naïve age-matched control animals (Control) from the same population.

Anesthesia was induced with 6 % sevoflurane for 4 min in a box. Then the rats were intubated with an adapted venflon (14GA venflon™ BD Luer-Lok™, Helsingborg, Sweden) and mechanically ventilated with 65/min and 3.5 cm^3^, 21 % O^2^ (Havard® Inspira advanced safety ventilator, volume controlled, MA1 55–7058, Holliston, Massachusetts, USA). To maintain anesthesia during preparation, 3.5 % sevoflurane was used and buprenorphine 20 μg/1000 g was administered subcutaneously before start of preparation. Temperature probes (General Purpose Sensor 9F, Mon-a-therm™, A Mallinckrodt Company, Mexico) were advanced into the esophagus (Tes) and rectum (Trec). Baseline Tes was maintained at 37 ± 0.2 °C with a heated operating table for small animals (Medax GmbH & Co, Neumünster/Germany). Two catheters (Argyle™ Polyurethane Umbilical Vessel Catheter; 2.5 Fr, Convidien™, Mansfield, USA) were inserted via cut down 11 and 9 cm via the left femoral vein and artery for hemodynamic monitoring, blood sampling and for administration of medications and infusions. A pacing catheter (Vygon GmbH & Co Bi-Pacing-ball 3 Fr, Aachen, Germany) was inserted in the right jugular vein via cut down for inducing ventricular fibrillation cardiac arrest during the experiment. After cardiopulmonary parameters were stable the operating table was removed; the rat was transferred to a glass platter and restrained with tape across the extremities in a way that the thorax did not move during chest compression.

### Experimental protocol

Anesthesia was discontinued 1.5 min before the start of the experiment. Mechanical ventilation was stopped and ventricular fibrillation cardiac arrest induced with an external current impulse of maximum 12 mA for 2 min via the pacing catheter. After 8 min of ventricular fibrillation cardiac arrest, ventilation (100 % oxygen, 40/min) and mechanical chest compressions were initiated at 200 beats per minute with a small animal resuscitator (Streubel Automation, Grampersdorf, Germany). At 2 min of cardiopulmonary resuscitation, rats were defibrillated 2 times with 5 J; biphasic and defibrillation attempts repeated every 2 min (Phillips MRX Defibrillator with adapted defibrillator pads, Andover, MA 01810–1085, USA). Epinephrine 20 μg/kg and bicarbonate 1 mmol/kg were given iv 1 min prior to the start of cardiopulmonary resuscitation. Epinephrine alone at 10 μg/kg was administered 75 s after the start of cardiopulmonary resuscitation, and repeated every 2 min during cardiopulmonary resuscitation. If restoration of spontaneous circulation was not achieved 12 min after initiation of cardiopulmonary resuscitation, the experiment was terminated. Upon achieving spontaneous circulation, catheters were removed, cut downs surgically treated and the animals weaned off mechanical ventilation. Animals were provided with oxygen and heating lamps to keep them normothermic. Analgesia was maintained with buprenorphine 12 μg/500 g as long as pain was observed. As soon as obvious normal recovery was achieved, animals were kept in groups of 4 in cages.

### Morris Water Maze

**Fig. 1:**
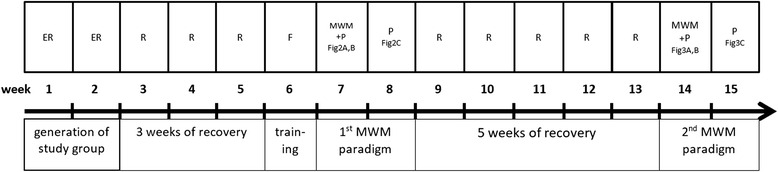
Experimental schedule: each rectangle on top of the timeline representing one week of the experimental protocol; each rectangle under the timeline representing an experimental phase of this study. *ER* Experimental Resuscitation Week, *R* Recovery Week, *F* Familiarization Week, *MWM* + *P* Morris Water Maze and Probe Trial

After the experiment in week 1 and 2 of the schedule (Fig. 1) each animal had at least 3 weeks to allow for complete recovery. After this recovery phase the animals had 1 week of familiarization (in week 6 of the schedule). Daily 15 min intensive contact with the investigator including feeding by hand, climbing exercises on the investigator and training on a wooden beam of 1.5×1.5 cm diameter and 2 m length via which the animal’s cage was reachable, were performed. This test was adapted from the Beam Balance Test [19] and Beam Walking Test. [20] On the last day of this pre-testing week, the time needed for the distance of 1 m on this beam was measured three times and the shortest time recorded. A time less than 4 s was considered to be motorically qualified for Morris Water Maze testing in the following week.

For said testing, a water tank of 2.20 m diameter and 60 cm height made of black plastic filled with water at 22 °C to the height of 30 cm was used and placed in a small room. Data recording was performed with a computer system for behavioral testing (Noldus, EthoVision, Wageningen, Netherlands) in combination with a video camera placed in the center of the ceiling of the room. Visual landmarks different in sizes and colors were placed on 3 walls, and a green cloth was stretched across the 4 side of the tank to hide the investigator and the computer system. The room had dimmed light which did not reflect on the water surface directly but was enough to enable the animals to see the walls around the water tank. The tank was divided into 4 quadrants (NW, NE, SW, SE) and in one of these a platform (10×10cm; height 28 cm; black) was positioned 2 cm under the water surface 20 cm away from the edge of the tank and attached to the floor. Finding this invisible platform hidden under the water surface by orienting themselves via visual cues on the walls made it possible for the animals to escape the cold water.

In week 7 of the experimental schedule each morning animals were tested in the Morris Water Maze which was 5 weeks after cardiac arrest, using the paradigm 1 consisting of 3 trials (each a maximum of 120 s) per day (with varying starting points) over 4 consecutive days (acquisition). After locating the platform on each trial, animals remained there for 30 s and were removed from the pool. In the case that the platform was not found in 120 s, the rat was guided on the water surface to reach the platform where it then remained for 30 s. The rats rested at least 15 min between swimming exercises, were dried with towels by hand and released into their cages until the next run. A probe trial following removal of the platform was conducted 24 h after the last trial (55 s) and a retest was performed 7 days later in week 8 of the schedule for long-term memory. Time spent in the sector where the platform has been, was compared to the time spent in the other sectors. Rats were placed in the pool in locations chosen by means of a pseudo-random plan in all trials and probe paradigm. A second Morris Water Maze paradigm was used 7 weeks after the first paradigm in week 14 of the schedule consisting of only 2 trials per day (with varying starting points, each a maximum of 90 s) over 4 consecutive days. To prevent the animals to recall the first paradigm the visual cues have been relocated entirely. A probe trial and a repeated probe trial 7 days later in week 15 of the experimental schedule were performed.

### Statistics

We report continuous variables as means ± standard error of mean (SEM) and categorical variables as counts and percentages. Differences between and within groups have been assessed by Student T- test or two-way ANOVA followed by Scheffe’s post hoc comparisons, as appropriate. Wilcoxon rank-sum test was used to analyze the probe trials. PASW Statistics (version 20, SPSS, IBM Corporation, Somers, NY) was used. A two-sided *p*-value of less than 0.05 was considered statistically significant.

## Results and discussion

Restoration of spontaneous circulation was achieved in 15 rats, no restoration of spontaneous circulation in 8 rats and 4 experiments had to be excluded because of technical failures. During the first days of recovery, 3 of the rats with restoration of spontaneous circulation died due to multiple organ failure. Finally, 12 rats survived with good neurological recovery. Before the start of the Morris Water Maze testing, the rats weighed 357 ± 20 g in the resuscitated CA group and 374 ± 30 g in the Control group (*p* = 0.9, Student *T*-test).

One week before the start of the Morris Water Maze testing procedure, rats were investigated as to their ability to climb over a horizontally positioned wooden beam (1.5×1.5 cm) back to their cages as an indicator of normal motoric skills. The animals of the resuscitated CA group needed 4.1 ± 2.7 s whereas the Control group 2.3 ± 0.4 s (*p* = 0.011, Student *T*-test). The data of three animals, requiring more than 4 s for the test distance had to be excluded from the Morris Water Maze analysis because of obvious motoric constraints. Thereafter both groups were equal in their motoric performance on the beam (2.8 ± 0.7 vs. 2.3 ± 0.4 s; *p* = 0.521, Student *T*-test).

**Fig. 2:**
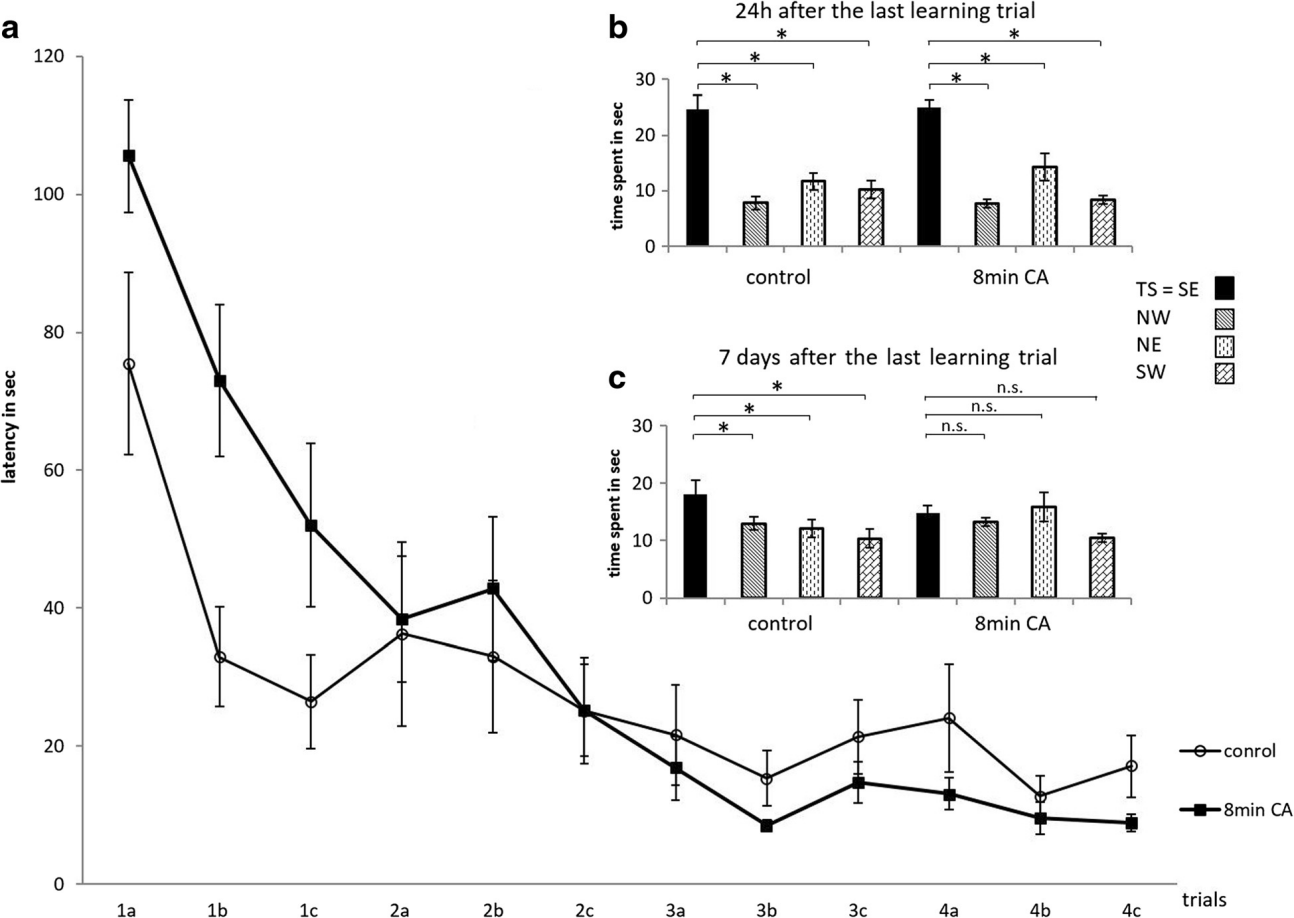
**a** Time needed to find the hidden platform 5 weeks after cardiac arrest in 8 min (min) cardiac arrest (CA) study group (*n* = 9); control, untreated control group (*n* = 10). Mean time ± SEM in seconds (sec) to find the hidden platform in this first Morris Water Maze paradigm with 3 trials (maximum time 120 s) daily (*a*, *b*, *c*) on 4 consecutive days (1–4). **b** and **c** Probe trial 24 h (**b**) and 7 days (**c**) after the last trial; mean time in seconds ± SEM (p values target sector vs each other quadrant * *p* < 0.05, *n.s*. not significant). *TS* target sector = *SE* south east sector, *NW* north west sector, *NE* north east sector, *SW* south west sector

*Five weeks after cardiac arrest* on 4 consecutive days, animals had 120 s 3 times daily in the Morris Water Maze to find the hidden platform under the water surface. The time to find the platform shortened from 105.6 ± 8.2 s in the CA arrest group and from 75.5 ± 13.2 s in the Control group in the 1st trial on day 1 to 8.9 ± 1.2 and 17.1 ± 4.5 s in the 3rd trial on day 4. W*ithin each group equally satisfying learning behavior in finding the hidden platform over 4 days was found* (*CA*: *p* < *0.001*, *Control*: *p* < *0.001*; Wilcoxon rank-sum test) *with no overall differences between groups* (*p* = *0.138*, *two*-*way ANOVA*). *However only on day 1* (*1a*, *b*, *c*) *the Control group showed a trend to need less time to find the hidden platform compared to the CA group* (*p* = *0.085*; Wilcoxon rank-sum test) (Fig. 2a). Twenty four hours after the last trials, the hidden platform was removed and animals’ searching behavior was recorded for 55 s by the computer system. The time spent in the target sector, where the platform has been in the trial days was compared with the time spent in the other 3 sectors. The animals of both groups spent a statistically significantly longer amount of time in the target sector (CA: 25 ± 1.3; Control: 24.7 ± 2.5 s) than in each of the three other sectors (CA: 7.7 ± 0.7, 14.3 ± 2.5, 8.4 ± 0.8 and Control: 7.8 ± 1.2, 11.7 ± 1.5, 10.3 ± 1.6 s; *p* < 0.05) but with no significant differences between groups (Fig. 2B). Seven days later the probe procedure was repeated. Resuscitated animals spent 14.8 ± 0.8 s in the target sector and 13 ± 0.8 (*p* = 0.343), 15.9 ± 1.1 (*p* = 0.635) and 10.5 ± 1 s (*p* = 0.066) in the other sectors (*p* values for target sector vs other sector) reflecting reduced long-term memory, whereas the Control group animals spent 18 ± 0.7 s in the target sector and 13.8 ± 1.6 (*p* = 0.047), 12.1 ± 1.2 (*p* = 0.007) and 10.4 ± 0.9 s (*p* = 0.005) in the other sectors (Fig. 2c).

**Fig. 3:**
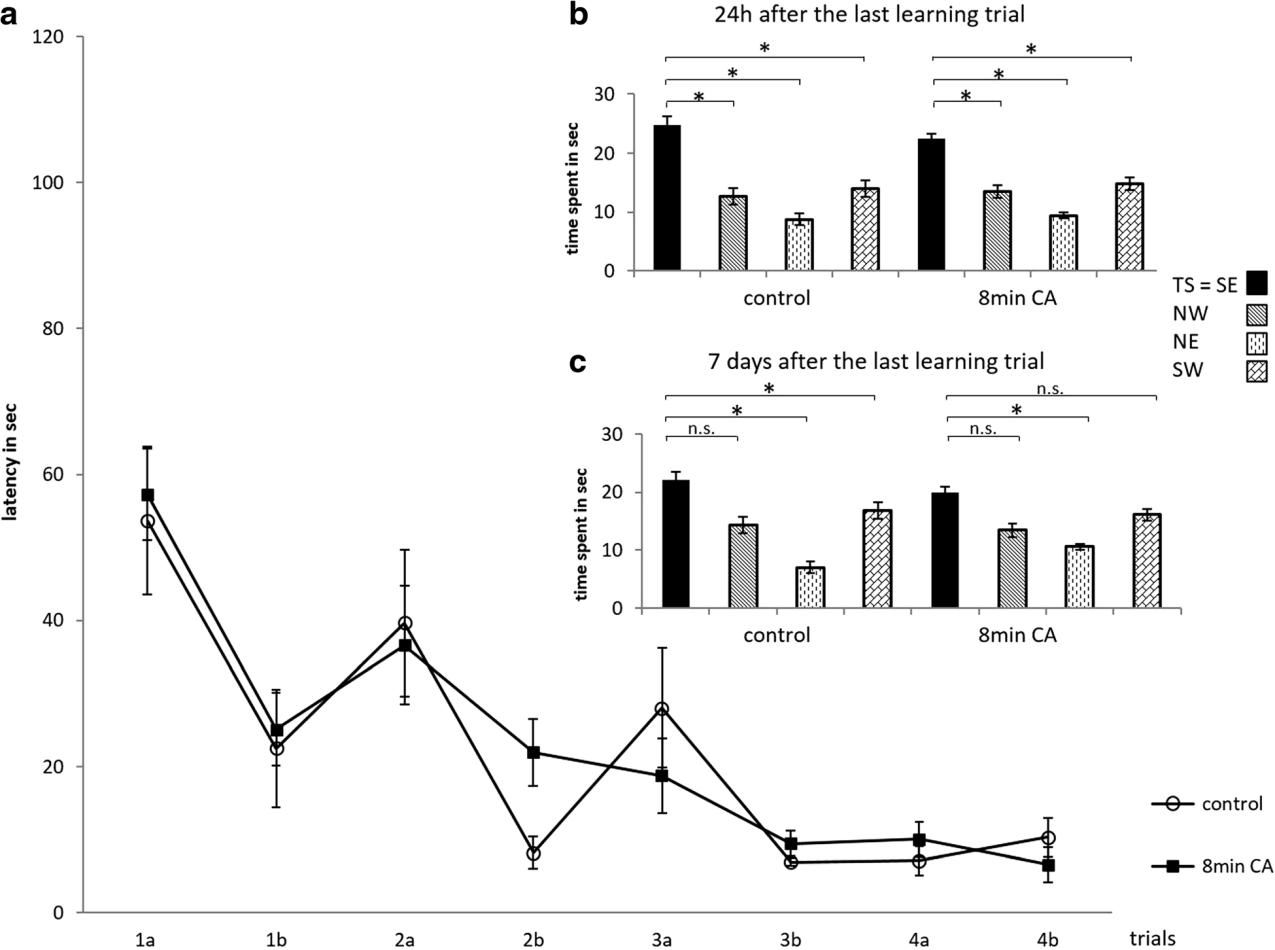
**a** Time needed to find the platform 12 weeks after cardiac arrest; second Morris Water Maze paradigm 7 weeks after first paradigm; 8 min (min) cardiac arrest (CA) study group (*n* = 12); control, untreated control group (*n* = 10). Mean time in seconds (sec) ± SEM to find the hidden platform in 2 trials (maximum time 90 s) daily (a, b) on 4 consecutive days (1–4). **b** and **c** Probe trial 24 h (**b**) and 7 days (**c**) after last trial; mean time in seconds ± SEM (p values target sector vs each other quadrant * *p* < 0.05, *n.s*. not significant). *TS* target sector = *SE* south east sector, *NW* north west sector, *NE* north east sector, *SW* south west sector

In the second Morris Water Maze paradigm 7 weeks after the first one e.g. 12 weeks after cardiac arrest the time to find the platform shortened from 57.3 ± 6.3 s in the CA group (*n* = 12, now 12 instead of 9 because the former 3 excluded fully recovered) and 53.7 ± 10.1 s in the Control group (*n* = 10) in the 1st trial on day 1 to 10.1 ± 2.4 and 10.3 ± 2.6 s on the 2nd trial on day 4. *Within each group equally satisfying learning behavior in finding the hidden platform over 4 days was found* (*CA*: *p* = *0.008*, *Control*: *p* = *0.003*; Wilcoxon rank-sum test) *with no overall and daily differences between groups* (*p* = *0.751*, *two*-*way ANOVA*) (Fig. 3a). Twenty four hours after the learning trials, the probe trial was carried out as previously for 55 s. Animals of both groups spent a longer time in the target sector than in the other quadrants (CA: 22.5 ± 0.9 s as compared to 13.5 ± 1.1 (*p* = 0.01), 9.4 ± 0.5 (*p* = 0.002) and 14.8 ± 1.0 (*p* = 0.005); Control: 24.8 ± 1.5 as compared to 12.7 ± 1.4 (*p* = 0.005), 8.7 ± 1 (*p* = 0.005) and 14 ± 1.4 (*p* = 0.007) respectively; *p* values for target sector vs other sector) (Fig. 3b). Seven days later the probe procedure was repeated. Cardiac arrest and control group showed long-term memory performance with 20 ± 1.8 and 22 ± 1.9 s in the target sector respectively (*p* = 0.512) and compared to the other sectors for the CA group 13.4 ± 1.1 s (*p* = 0.108), 10.6 ± 0.9 (*p* = 0.025), 16.1 ± 1 (*p* = 0.347) and for the control group 14.3 ± 2.1 (*p* = 0.074), 7 ± 1.3 (*p* = 0.007), 16.8 ± 1.4 (*p* = 0.022) (Fig. 3c). Thus, in the Morris Water Maze paradigm performed 7 weeks after the first paradigm both groups showed adequate learning behavior and short-time memory, however long-term memory showed a trend to be better in rats previously subjected to cardiac arrest.

This study showed that the Morris Water Maze could be a suitable additional tool for investigation of behavioral and cognitive impairments in rats resuscitated from 8 min of untreated ventricular fibrillation cardiac arrest. Six weeks after cardiac arrest and resuscitation, untreated animals showed better memory retention than resuscitated rats. Our results showed better performance of naïve rats in the second probe trial repeated 7 days after Morris Water Maze trials and in a second Morris Water Maze paradigm performed 12 weeks after cardiac arrest and resuscitation and 7 weeks after the first Morris Water Maze paradigm. Both groups performed equally in learning, but untreated animals showed better memory retention than resuscitated rats.

The Morris Water Maze as a behavioral testing paradigm in our model of ventricular fibrillation cardiac arrest has not yet been implemented. Learning curves show that all investigated animals performed well in the acquisition phase, meaning that both rats in the cardiac arrest group and naïve animals were able to learn the task. To eliminate bias in the Morris Water Maze, variation of the starting points each day forced animals to orientate themselves with the help of landmarks on the walls of the experimenting room. We have chosen this behavioral testing method for our pilot study to find new outcome strategies in resuscitation experiments with rats to focus on additional outcome parameters, comparable to the problems of human long-time cardiac arrest survivors.

Morris Water Maze data of 3 animals with obvious motoric deficits caused by the surgical intervention in the course of the experiment were excluded from the 5 week trial, because of poor results from an adapted beam test performed before the swimming paradigm [19, 20]. However, after 12 weeks these animals exhibited an entire recovery like that of the other animals and therefore were included in the 12 weeks testing. We tried to avoid all other disturbing influences on the sensitive Morris Water Maze paradigm by having the test performed by only one investigator in an as quiet as possible environment at the same time of day each time.

Animal handling and accustomization of the animals to the investigator is of essential importance. One week before the Morris Water Maze each animal of the cardiac arrest and control group was handled daily for 15 min by the investigator. During that time animals were fed by hand and got used to being manipulated and touched. Both groups were treated equally but needless to say, the cardiac arrest group was handled more often by the investigator due to the intensive care phase following cardiac arrest. To achieve statistically comparable results and homogeneous groups, all animals should be tested in the same experimental week. Animal models of cardiac arrest and resuscitation are sophisticated and all procedures are time intensive. There was a need to find a compromise between the time needed for generating enough surviving animals and the Morris Water Maze logistics. Therefore we were only able to pool our experimental group of 12 surviving animals for the Morris Water Maze within 2 weeks. Thus the group size was dependent on the achievable animals within the permissive time period for the Morris Water Maze paradigm. All of the animals received a rehabilitation period of 3 week, followed by 1 week of habituation with intensive handling as described.

Results of the first Morris Water Maze trials showed a trend to decreased learning performance in the cardiac arrest group on day one and two. Meanwhile, the control group learned to reach the hidden platform faster within the first 2 days; thereafter this difference between the groups decreased and on the last day both groups performed equally (Fig. 2a). After removal of the platform, both groups spent statistically significantly more time in the target sector than in the other three sectors, which means that all animals learned the task (Fig. 2b). One week later the cardiac arrest group showed an impaired memory recall, as suggested by the time spent in the targeted quadrant. Indeed, the treated group spent in average the same amount of time in each quadrant (n.s. in Fig. 2c), whereas the control group spent still significantly more time in the quadrant where the platform was. Morris Water Maze testing was repeated 12 weeks after cardiac arrest, 7 weeks after the first Morris Water Maze paradigm. During the second Morris Water Maze procedure, animals had to perform only two trials a day and the time was shortened from 120 to 90 s. With this change - reduced time and number of daily trials - the authors hoped to provoke a more significant difference between the study group and naïve controls by making it harder to learn in which sector the platform was hidden under the water surface. However, our results showed no difference between the groups in learning performance, neither in the acquisition phase nor in memory recall during the probe trials on day 5 (Fig. 3a and b) One week later, all the rats including controls and cardiac arrest group did not spend a significantly longer period of time in the target sector (Fig. 3c). This might be caused by the influence of increasing age of animals on learning and memory behavior as reported by Morris [14] and D’ Hooge [21] or might be explained by factors reported by Langdon et al. [22] who suggest a spontaneous repopulation of CA1 cells weeks after the ischemic insult. Histological outcome of these animals will be investigated and described in detail in a further publication. Langdon et al. exposed rats to 10 min of bilateral carotid artery occlusion and systemic hypotension, and showed significant long-term deficits in ischemic animals’ learning, memory (T-maze, radial arm maze), working memory (radial arm maze), and reference memory (Morris Water Maze, radial arm maze) abilities. With the Morris Water Maze, we have chosen a well-known behavioral testing method as a first step for our model.

In resuscitation experiments it is difficult to minimize influencing factors. The animals cannot be resuscitated all on 1 day, they will differ in weight depending on their recovery processes; they underwent surgical inventions with different courses of healing. Recovery is an individual process like in human patients. The experiment procedure and the recovery phase may be very stressful and influence on performance in the water maze is hard to estimate. All these factors together show the limitations of this kind of investigation in a ventricular fibrillation cardiac arrest model.

## Conclusions

Spatial learning and memory recall in clinically intact animals without neurological signs following resuscitation from 8 min ventricular fibrillation cardiac arrest was comparable to normal healthy controls. However, memory recall after a 7 day period without training was impaired. In a second reduced Morris Water Maze paradigm 12 weeks after cardiac arrest, animals performed equally in the control and study groups. It could be shown that memory retention rather than learning and memory recall is valuable after global ischemia in this particular animal model for cardiac arrest. Our findings point toward a need for further modification of the Morris Water Maze paradigm in order to target memory retention or for finding more fitting behavior testing setups to suit the particulars of resuscitation models.

## Abbreviations

CA, cardiac arrest; CA1, cornu ammonis 1 region; Tes, esophageal temperature; Trec, rectal temperature; ER, experimental resuscitation week; R, recovery week; F, familiarization week; MWM + P, Morris Water Maze and Probe Trial; TS, target sector; SE, south east sector; NW, north west sector; NE, north east sector; SW, south west sector; n.s., not significant
